# Profiles of motivational impairment and their relationship to functional decline in frontotemporal dementia

**DOI:** 10.1007/s00415-024-12430-0

**Published:** 2024-05-17

**Authors:** Siobhán R. Shaw, Kristina S. Horne, Olivier Piguet, Rebekah M. Ahmed, Alexis E. Whitton, Muireann Irish

**Affiliations:** 1 Brain and Mind Centre, The University of Sydney, Sydney, NSW Australia; 2 School of Psychology, The University of Sydney, Sydney, NSW Australia; 3 Memory and Cognition Clinic, Department of Clinical Neurosciences, Royal Prince Alfred Hospital, Sydney, NSW Australia; 4 Black Dog Institute, University of New South Wales, Sydney, NSW Australia

**Keywords:** Motivation, Apathy, Anhedonia, Functional impairment, Younger-onset dementia, Neuropsychiatry

## Abstract

Motivational disturbances are pervasive in frontotemporal dementia (FTD) and impact negatively on everyday functioning. Despite mounting evidence of anhedonia in FTD, it remains unclear how such changes fit within the broader motivational symptom profile of FTD, or how anhedonia relates to functional outcomes. Here we sought to comprehensively characterize motivational disturbances in FTD and their respective relationships with functional impairment. A cross-sectional study design was used including 211 participants—68 behavioral-variant FTD (bvFTD), 32 semantic dementia (SD), 43 Alzheimer’s disease (AD), and 68 healthy older control participants. Anhedonia severity was measured using the Snaith–Hamilton Pleasure Scale while severity of apathy was assessed across Emotional, Executive, and Initiation dimensions using the Dimensional Apathy Scale. Functional impairment was established using the FTD Functional Rating Scale (FRS). Distinct motivational profiles emerged in each dementia syndrome: a domain-general motivational impairment in bvFTD; a predominantly anhedonic profile in SD; and more pronounced initiation and executive apathy in AD. Correlation analyses revealed differential associations between motivational symptoms and severity of functional impairment in each group. Executive apathy was associated with functional impairment in bvFTD, while anhedonia was strongly correlated with functional decline in SD. Finally, executive and emotional apathy were associated with functional decline in AD. Our study indicates distinct profiles of apathy and anhedonia in FTD syndromes, which in turn are differentially associated with functional decline. This detailed characterization of motivational phenotypes can inform patient stratification for targeted interventions to improve functional outcomes.

**Supplementary Information:**

The online version contains supplementary material available at 10.1007/s00415-024-12430-0.

## Introduction

Motivational disturbances are prominent in frontotemporal dementia (FTD), leading to social withdrawal and disengagement from previously enjoyed activities [1]. Loss of motivation in the form of apathy represents a core clinical feature of the behavioral variant of FTD [bvFTD; 2] and is increasingly recognized in language variants of FTD [3, 4]. Apathy is inherently multidimensional and can be deconstructed in terms of its impact on goal-oriented behavior, cognitive engagement, or emotional responsiveness [5, 6]. Unsurprisingly, apathy is a strong predictor of functional decline in FTD [7, 8], compromising the organization, planning, and initiation of basic activities of daily living including cooking, managing medication, and maintaining personal hygiene [8–10]. Beyond its impact on the individual, apathy has downstream negative effects on the social and emotional wellbeing of the carer [1, 11, 12].

While apathy has tended to take center stage, recent studies suggest that many of the negative behavioral symptoms of FTD (e.g., social withdrawal, lack of engagement, loss of interest) might be better conceptualized in terms of alterations in hedonic processing [13–15]. Anhedonia refers to the loss of interest in previously enjoyable pursuits, such as food, sex, hobbies, and social interactions, and is pervasive in neuropsychiatric disorders, such as depression and bipolar disorder [16]. Several recent studies demonstrate the presence of marked anhedonia in bvFTD and semantic dementia (SD), representing a significant departure from pre-morbid functioning [16–18]. Importantly, while anhedonia and apathy co-occur in FTD, their neural substrates are dissociable, suggesting that anhedonia should be considered a standalone clinical symptom in FTD [16]. These observations have prompted calls for a more targeted approach to identifying distinct motivational phenotypes in FTD [18, 19].

Despite growing awareness that apathy and anhedonia co-occur in FTD, their differential impact on functional impairment in FTD syndromes remains unclear. Studies in psychiatric disorders, such as major depressive disorder, schizophrenia, and bipolar disorder, suggest a close coupling between anhedonia, negative functional outcomes, and disease severity [20, 21]. However, no study to date has explored the impact of anhedonia on functional outcomes in FTD. The objective of the present study therefore was to: (1) characterize the nature of multidimensional motivational impairments in bvFTD and SD compared to Alzheimer’s disease (AD) participants; and (2) explore the relative contributions of multidimensional apathy and anhedonia to functional impairment in each diagnostic group.

## Methods

### Participants^1^

A total of 211 participants were recruited through FRONTIER, the frontotemporal dementia research clinic based at the Brain and Mind Centre, at the University of Sydney, Australia. Within this group, 68 participants met current diagnostic criteria for clinically probable bvFTD [2], 32 met criteria for SD [21 left- and 11 right-lateralized; 22] and 43 participants met criteria for typical AD [23]. Participant diagnoses were established by consensus among a multidisciplinary team including a senior neurologist (R.M.A.), neuropsychologist, and occupational therapist based on comprehensive clinical investigation, neuropsychological assessments, informant interview, and review of brain atrophy on structural MRI.

Sixty-eight healthy older control participants (age > 55 years) were recruited from the FRONTIER volunteer database and local community groups. All controls scored > 88 on the Addenbrooke’s Cognitive Examination III screening tool [ACE-III; 24, 25], zero on the Clinical Dementia Rating Scale [26], and performed within normal limits on all behavioral and cognitive measures.

Exclusion criteria for all participants included a prior history of mental illness, alcohol or other drug abuse, significant head injury, and limited English language proficiency. Patients scoring < 40 on the ACE-III (max score = 100) were excluded due to the severity of their cognitive impairment.

### Clinical and cognitive assessment

The ACE-III was used to determine participants’ overall level of cognitive function across Attention and Orientation, Memory, Fluency, Language, and Visuospatial function [24, 25]. Behavioral changes were assessed using the carer-rated Cambridge Behavioural Inventory-Revised [CBI-R; 27]. Disease duration was calculated as the number of years elapsed from reported onset of symptoms to date of testing. The depression subscale of the Depression, Anxiety, and Stress Scale [DASS; 28] was used to assess participants’ self-reported mood over the past week. For patients, each item was read aloud by the clinician and wording was clarified where necessary.

### Carer-rated assessment of motivational disturbances

Carers rated levels of apathy and anhedonia in the patients across two time periods (“Before symptom onset” and “Current”). Carers predominantly comprised spouses or partners (80%) and lived at home with the patient, providing a reliable index of pre-morbid and current levels of motivation. A full breakdown of carer characteristics is provided in Supplementary Material.

### Apathy

The Dimensional Apathy Scale (DApS; [29]) was included as a validated carer-rated assessment of apathy (1) before dementia onset and (2) at the current time [29]. The DApS comprises three subscales assessing disrupted planning, attention, and organization (Executive); blunted emotional responses (Emotional); and loss of spontaneous activity (Initiation). Carers rate each item on a 4-point Likert scale based on the frequency of occurrence in the last month from 0, ‘Hardly ever’ to 3, ‘Almost always’ (max score: 24 per subscale).

### Anhedonia

The Snaith–Hamilton Pleasure Scale [SHAPS; 30] was used to index carer ratings of the capacity to experience pleasure in the patient. Scores range from 14 to 56, with lower scores indicating greater anhedonia. We modified the SHAPS to probe carer ratings of patient anhedonia across two time points: (1) before dementia onset and (2) current time. The SHAPS has previously been shown to be sensitive to changes in hedonic tone in the three dementia syndromes of interest [for full details see 16].

### Functional impairment

The Frontotemporal Dementia Functional Rating Scale [FRS; 31] was used as a validated index of carer-rated functional impairment and disease staging. The FRS is a 30-item questionnaire probing the frequency of difficulties that patients experience (e.g., ‘all the time’, ‘sometimes’, ‘never’) across seven domains in their daily lives. Lower scores denote greater functional impairment.

### Statistical analyses

Statistical analyses for cognitive and clinical data were performed using IBM SPSS Statistics, version 27.0. Normality of distributions was assessed via visual inspection of boxplots and Shapiro–Wilk tests (see Supplementary Material). Where variables were normally distributed, separate univariate analyses of variance (ANOVAs) were used to examine group differences on continuous demographic variables (e.g., age, education) with Sidak post hoc tests. Chi-square tests were used to investigate group differences on categorical variables (e.g., sex).

To compare across scales, an index of dementia-related changes in motivation was calculated based on carer ratings of pre-morbid (i.e., before disease onset) compared to current (i.e., time of assessment) levels of apathy and anhedonia. Patient and control groups did not differ in terms of pre-morbid ratings on either the SHAPS or DApS (see Supplementary Material). Therefore, separate linear regression models were run with pre-morbid ratings included as the predictor and current ratings included as the dependent variable [32]. Standardized residuals were then extracted for each patient for the SHAPS and DApS and these residual scores were used as an index of disease-related anhedonia and apathy severity, respectively. Positive residual scores indicate an increase in severity (i.e., greater apathy or anhedonia), while negative residual scores represent a decrease in severity since dementia onset.

To determine whether profiles of apathy and anhedonia differed across diagnostic groups, we ran a 3 × 4 repeated measures ANCOVA controlling for sex, disease duration, and overall level of cognitive function on the ACE-III. Here, we explored main effects of Group (AD, bvFTD, SD), and Domain (anhedonia, executive apathy, emotional apathy, initiation apathy), as well as relevant interactions, on apathy and anhedonia residual scores. Significant interactions and/or main effects were followed up with Sidak post hoc tests. Partial eta-squared values ${\eta }_{{\text{p}}}^{2}$ accompany all ANOVAs and ANCOVAs as measures of corresponding effect sizes.

Partial Pearson correlations were run in each patient group to examine associations between motivational severity scores and functional impairment (FRS), controlling for sex, disease duration, and overall level of cognitive dysfunction on the ACE-III. To correct for multiple comparisons, the Benjamini–Hochberg procedure was used [33], with a critical alpha level of 0.05. Finally, Fisher’s *r* to *Z* transformations were used to determine which variables more strongly predicted functional impairment within each group.

## Results

Demographic, cognitive, and clinical characteristics of the study sample are presented in Table 1. Groups were not statistically significantly different in terms of age [*F*(3,207) = 2.4, *p* = 0.07, ${\eta }_{{\text{p}}}^{2}$ = 0.03] but differed for sex distribution (*χ*^*2*^ = 13.93, *p* = 0.003) driven by significantly more males in the bvFTD group and females in the control group (both *p*’s < 0.05). Groups also differed for years of education [*F*(3,207) = 4.5, *p* = 0.005, ${\eta }_{{\text{p}}}^{2}$ = 0.06] as the bvFTD group had, on average, 1.9 fewer years of education relative to Controls (*p* = 0.004; all other *p*’s > 0.08). Patient groups had comparable disease duration (years elapsed since onset of symptoms; *p* = 0.09) but differed in terms of functional impairment on the FRS [FRS Logit score: *F*(2,127) = 3.9, *p* = 0.02, ${\eta }_{{\text{p}}}^{2}$ = 0.06], with bvFTD patients showing greater functional impairment relative to SD (*p* = 0.04). Importantly, using the FRS classification for disease staging, all dementia groups were categorized as being at the same level of disease severity (i.e., “moderate”). No other significant differences were observed on the FRS (all *p*’s > 0.09).

**Table 1:** Demographic, clinical, and cognitive characteristics of study cohort

	bvFTD (*n* = 68)	SD (*n* = 32)	AD (*n* = 43)	Control (*n* = 68)	Test statistic	Post hoc comparisons
Age [years]	64.6 (7.6)	67.9 (7.0)	66.2 (8.0)	67.5 (6.0)	*F* = 2.4	–
Education [years]	12.5 (3.3)	12.7 (3.2)	13.3 (3.0)	14.3 (3.2)	*F* = 4.5**	Control > bvFTD
Sex, M:F	47:21	14:18	26:17	27:41	*χ*^2^ = 13.9**	M > F
Disease duration [years]	6.4 (4.0)	6.5 (3.2)	5.0 (2.3)	n/a	*F* = 2.4	–
ACE-III Total [100]	76.0 (14.4)	60.7 (12.7)	62.3 (12.9)	95.4 (3.3)	*F* = 105.1***	Control > bvFTD > AD, SD
FRS Logit score^a^	− 0.21 (1.6)	0.64 (1.6)	0.47 (1.3)	n/a	F = 3.9*	SD > bvFTD
FRS Stage	Moderate	Moderate	Moderate	n/a	n/a	n/a
CBI-R Total [%]	37.4 (17.2)	31.0 (14.7)	30.5 (14.2)	n/a	*F* = 3.1	–
DASS Depression [21]	5.2 (4.7)	4.0 (4.5)	5.0 (4.5)	1.1 (1.5)	*F* = 13.7***	Patients > Controls

Values are in the format mean (standard deviation) unless otherwise specified. Maximum score and/or unit of measurement for each test shown in square brackets where appropriate

^a^Lower logit scores, as derived using Rasch analyses, denote greater levels of functional impairment on the FRS

*p < 0.05; **p < 0.005; ***p < 0.0001; – not significant, n/a not applicable, *ACE*-*III* Addenbrooke’s Cognitive Examination third edition, *AD* Alzheimer’s disease, *bvFTD* behavioral variant of frontotemporal dementia, *CBI*-*R* Cambridge Behavioural Inventory-Revised, *DASS* Depression, Anxiety and Stress Scale, *F* female, *FRS* Frontotemporal Dementia Functional Rating Scale, *M* male, *SD* semantic dementia. CBI-R missing for 2 SD, 2 bvFTD. DASS-D missing for 5 AD, 6 bvFTD, 6 SD and 9 controls. Disease duration missing for 4 AD, 4 bvFTD and 1 SD. FRS missing for 1 AD, 10 bvFTD and 2 SD

Group differences were evident on the DASS-D [*F*(3,185) = 13.7, *p* < 0.001, ${\eta }_{{\text{p}}}^{2}$=0.19] with patients self-reporting higher levels of depression relative to Controls (all *p*’s < 0.008). However, patient groups did not differ for self-rated depression (all *p*’s > 0.72) or carer-rated behavioral change on the CBI-R (all *p*’s > 0.09). Significant group differences were evident on the ACE-III [*F*(3,206) = 105.1, *p* < *0.0*01, ${\eta }_{{\text{p}}}^{2}$=0.61], driven by marked cognitive impairment in patients relative to Controls (all *p*’s < 0.001). Post hoc tests indicated that AD and SD patients were disproportionately impaired relative to the bvFTD group (both *p*’s < 0.001) with no difference between AD and SD (*p* = 0.99). These cognitive and behavioral changes are in keeping with previous studies.

### Disease-specific changes in severity of motivational disturbances

A repeated measures ANCOVA, controlling for sex, disease duration, and overall level of cognitive dysfunction on the ACE-III, revealed a significant main effect of Group [*F*(2,113) = 15.9, *p* < 0.001, ${\eta }_{p}^{2}$=0.32], where, irrespective of motivational domain, bvFTD patients showed greater motivational impairments than SD (*p* < 0.001) and AD (*p* < 0.001) patients. No difference was observed between the SD and the AD groups (*p* = 0.86). No main effect of Domain was observed [*F*(3,336) = 0.69, *p* = 0.56, ${\eta }_{p}^{2}$=0.02].

**Fig. 1:**
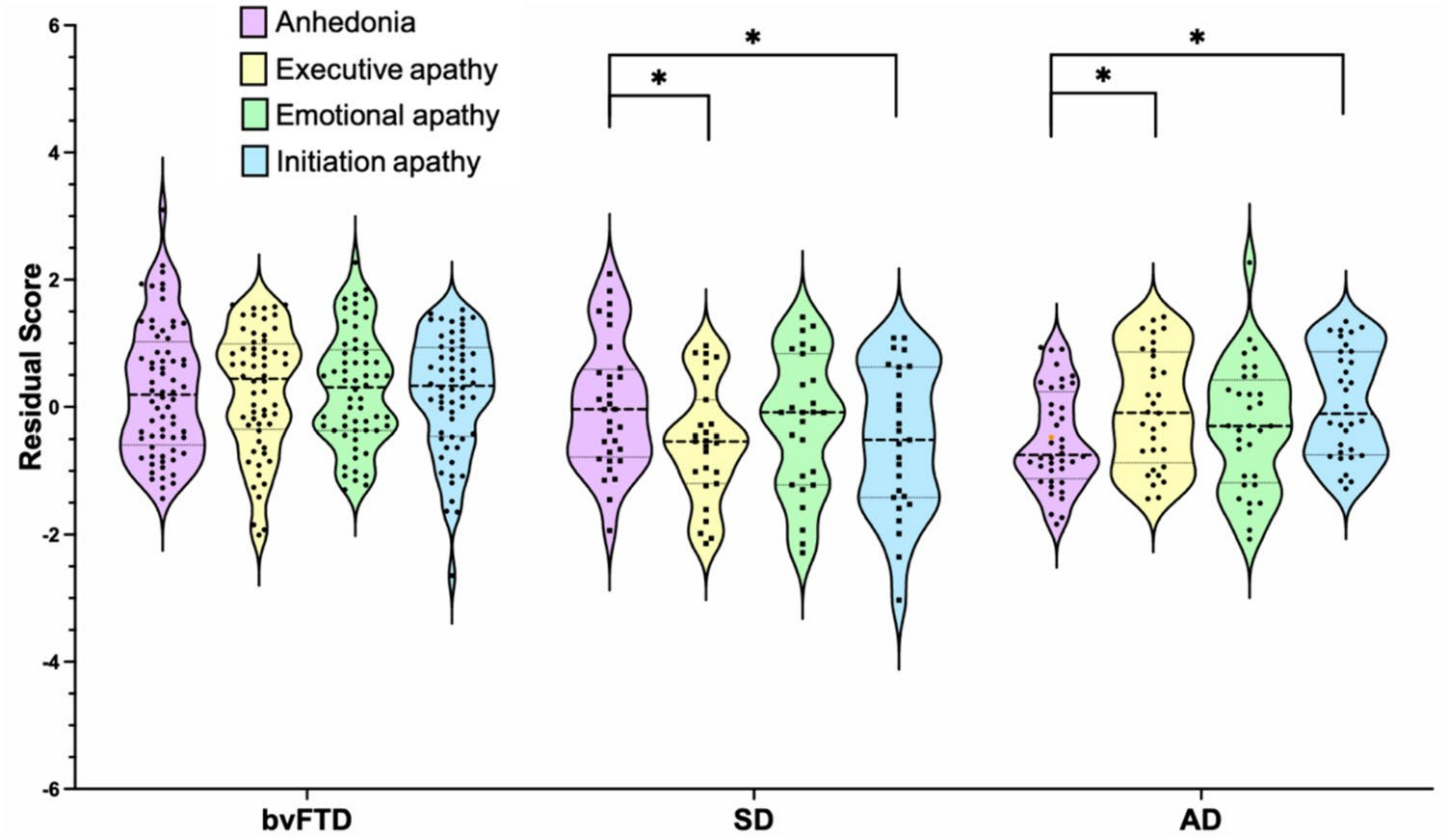
Disease-specific motivational profiles across dementia syndromes. Residual scores derived from separate linear regression models with pre-morbid (before dementia onset) carer ratings included as the predictor and current carer ratings included as the dependent variable for anhedonia (SHAPS) and apathy (DApS) severity. Violin plots depict the distribution of data with the width of the shaded area representing the proportion of data located there. Bolded horizontal line depicts the median, while dotted lines depict quartiles. *AD* Alzheimer’s disease, *bvFTD* behavioral variant of frontotemporal dementia, *SD* semantic dementia. Asterisks denote significant within-group differences. *p < 0.05

The Group by Domain interaction was significant [*F*(6,336) = 6.0, *p* < 0.001, ${\eta }_{p}^{2}$=0.10]. Post hoc tests investigating the simple effect of Domain revealed distinct motivational profiles in each group. All subscales were comparably affected in bvFTD (all *p*’s > 0.98) reflecting a domain-general motivational impairment. In contrast, for SD, the motivational profile was dominated by significantly higher levels of anhedonia relative to executive (*p* = 0.01, mean difference = 0.64, 95% CI 0.1–1.2) and initiation (*p* = 0.01, mean difference = 0.59, 95% CI 0.07–1.1) apathy, but with no difference compared to emotional apathy (*p* = 0.67). Finally, AD patients displayed higher executive (*p* = 0.009, mean difference = 0.61, 95% CI 0.11–1.11) and initiation (*p* = 0.004, mean difference = 0.62, 95% CI 0.14–1.1) apathy compared to anhedonia (see Fig. 1).

Sidak post hoc tests were run to assess the simple effect of Group within each motivational Domain. Anhedonia severity was not found to differ significantly between bvFTD and SD (*p* = 0.08), but was of a significantly greater magnitude in the FTD syndromes relative to AD (bvFTD mean difference = 1.11, 95% CI 0.60–1.63; *p* < 0.001; SD mean difference = 0.60, 95% CI 0.01–1.18, *p* = 0.04). Executive apathy was significantly higher in bvFTD relative to SD (*p* < 0.001, mean difference = 1.2, 95% CI 0.66–1.8) and AD (*p* = 0.03, mean difference = 0.57, 95% CI 0.66–1.8), while AD patients exhibited greater executive apathy relative to SD (*p* = 0.03, mean difference = 0.65, 95% CI 0.06–1.3). Emotional apathy was most pronounced in bvFTD relative to SD (*p* = 0.04, mean difference = 0.61, 95% CI 0.02–1.2) and AD (*p* = 0.003, mean difference = 0.77, 95% CI 0.22–1.3), with no difference between the SD and the AD groups (*p* = 0.88). Finally, initiation apathy was the greatest in bvFTD relative to AD (mean difference = 0.58, 95% CI 0.06–1.09, *p* = 0.02) and SD (mean difference = 1.2, 95% CI 0.66–1.1; *p* < 0.001), while AD patients showed higher initiation apathy relative to SD (mean difference = 0.66, 95% CI 0.06–1.2, *p* = 0.03). See Supplementary Material for further information.

Taken together, our findings indicate distinct motivational profiles in each patient group. BvFTD displayed the most profound motivational disturbances, spanning all motivational domains. The SD motivational profile was largely driven by hedonic deficits, with significantly lower levels of executive and initiation apathy. Conversely, the AD profile was characterized by initiation and executive apathy, albeit at a lower level than that observed in bvFTD, with AD patients showing the lowest level of anhedonia relative to the other groups.

### Relationship to functional impairment

**Fig. 2:**
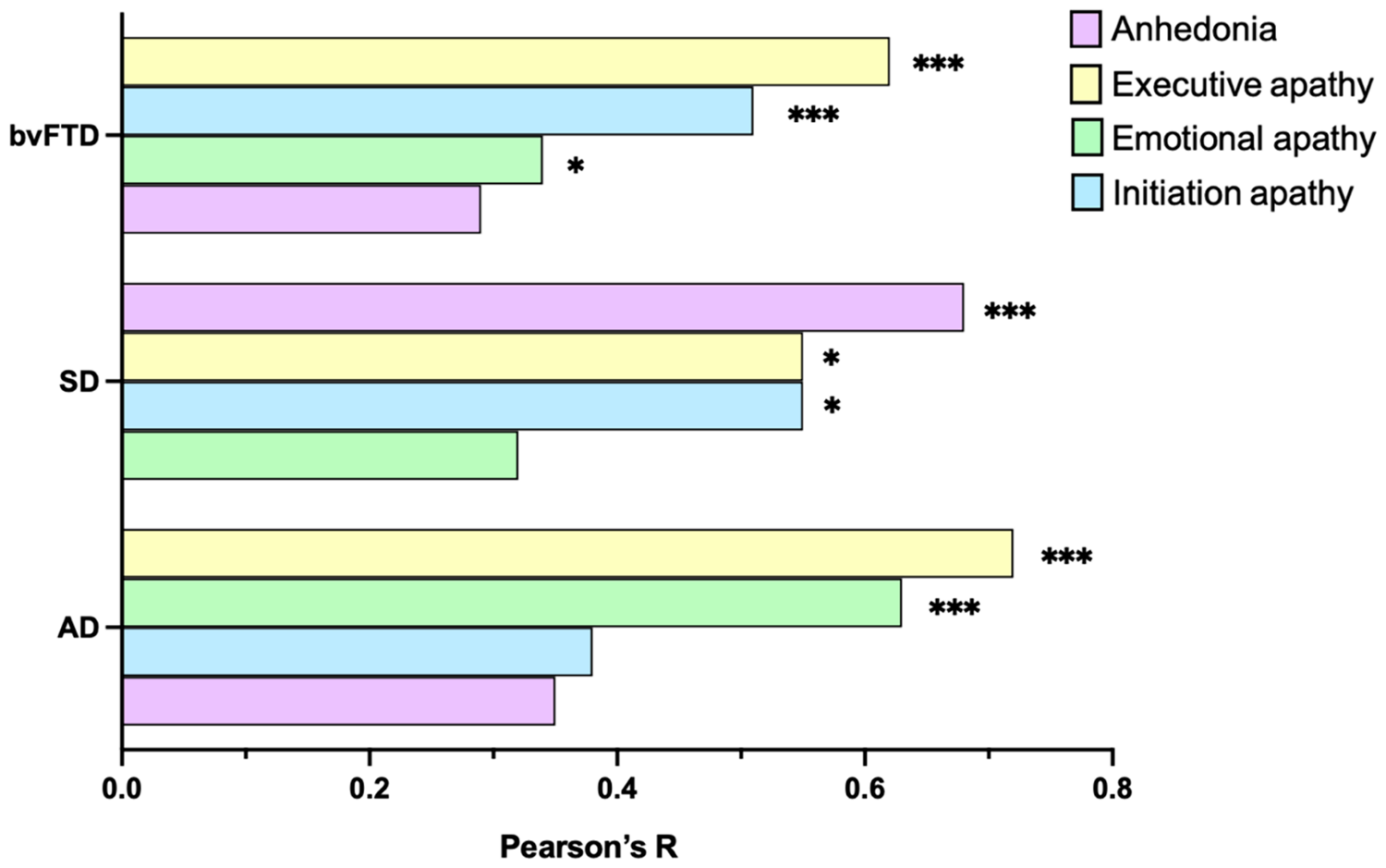
Relative associations between severity of motivational disturbances and functional impairment in dementia. Pearson r values presented as absolute values in ascending order representing the strength of association between severity of motivational impairment and functional decline in each patient group. *p < 0.05, ***p < 0.001. *AD* Alzheimer’s disease, *bvFTD* behavioral variant of frontotemporal dementia, *SD* semantic dementia

We next explored how severity of motivational changes relates to functional impairment within each patient group (Table 2). Importantly, neither disease duration (all *p*’s > 0.07) nor self-reported Depression on the DASS-21 (all *p*’s > 0.26) was found to relate to anhedonia or apathy in any of the patient groups. Figure 2 presents an overview of the relative associations between multidimensional motivational impairments and functional outcomes in each patient group (scatterplots presented in Supplementary Material).

**Table 2:** Associations between severity of motivational disturbances and functional impairment in each dementia group

		bvFTD (*n* = 58)	SD (*n* = 30)	AD (*n* = 42)
FRS	Anhedonia	− 0.29*	− 0.68***	− 0.35
	Executive apathy	− 0.62***	− 0.55*	− 0.72***
	Emotional apathy	− 0.34*	− 0.32	− 0.63***
	Initiation apathy	− 0.51***	− 0.55*	− 0.38

Bolded values represent correlations that remain significant following Benjamini–Hochberg correction for false discovery rate at *q* = 0.05. *p < 0.05, ***p < 0.001

*AD* Alzheimer’s disease, *bvFTD* behavioral variant of frontotemporal dementia, *FRS* Frontotemporal Dementia Functional Rating Scale, *SD* semantic dementia

Pearson partial correlations controlling for sex, disease duration, and overall level of cognitive dysfunction on the ACE-III revealed significant associations between functional impairment (FRS logit score) and all motivational domains in bvFTD, whereby more severe motivational disturbances were consistently related to greater functional impairment (all *p*’s < 0.03). Fisher’s *r* to *Z* transformations revealed that for bvFTD, executive apathy severity was more strongly related to functional decline than anhedonia (*Z* = 2.24, *p* = 0.01) or emotional apathy (*Z* = 1.95, *p* = 0.03), but did not differ from initiation apathy (*Z* = 1.45, *p* = 0.09).

In SD, functional decline was associated with all motivational changes (all *p*’s < 0.01), with the exception of emotional apathy (*p* = 0.16). Fisher’s *r* to *Z* transformations highlighted that anhedonia was more strongly correlated with functional impairment than emotional apathy (*Z* = 1.83, *p* = 0.03), with no other differences observed (all *p*’s > 0.28).

Finally, in AD, greater functional impairment was associated with severity of executive (*p* < 0.001) and emotional (*p* < 0.001) apathy. All other correlations were not significant (all *p*’s > 0.06). Fisher’s *r* to *Z* transformations indicated that executive apathy was more strongly correlated with functional impairment than anhedonia (*Z* = 2.39, *p* = 0.008) and initiation apathy (*Z* = 2.24, *p* = 0.01), but did not differ from emotional apathy (*Z* = 0.46, *p* = 0.32).

## Discussion

Loss of motivation is a prominent feature of FTD, yet systematic characterization of multidimensional changes in apathy and anhedonia has been lacking. Here, we provide a fine-grained profiling of phenotypic motivational disturbances and their respective contributions to functional impairment in FTD. Overall, we found distinct motivational profiles independent of disease severity in each dementia syndrome; a domain-general dampening of motivation in bvFTD, a predominantly anhedonic profile in SD, and an apathetic profile driven by pronounced initiation and executive apathy in AD. Importantly, dimensions of apathy and anhedonia were differentially related to functional outcomes in each patient group suggesting the need for targeted interventions geared toward the specific motivational profile of the individual.

Considering first the bvFTD group, we uncovered a profile of profound motivational impairments, spanning all apathy dimensions on the DApS (executive, emotional, initiation). These changes in goal-directed behavior were more severe relative to disease-matched cases of SD and AD, in keeping with previous studies [1]. In addition, anhedonia was prominent in bvFTD, again of a greater magnitude than reported in AD, but comparable with the SD group, consistent with previous findings [16]. Importantly, the magnitude of apathy and anhedonia severity was comparable across all subdomains in bvFTD, indicative of a global motivational impairment in this syndrome. Despite this overall dampening of motivation in bvFTD, correlation analyses suggested that executive apathy was more strongly associated with functional decline compared to emotional apathy and anhedonia, while initiation apathy was as strongly related to functional decline as executive apathy. Several studies in bvFTD have documented the negative impact of apathy, as a global construct, on instrumental activities of daily living, such as managing finances and shopping, as well as more advanced activities, such as playing games, planning, and going on holiday [reviewed by 8, 34]. Crucially, our findings caution against a simple one-to-one mapping between apathy and functional outcomes and suggest that a more fine-grained approach is needed. Notably, behavioral inertia and economy of cognitive effort appear particularly important for poor prognostic outcomes in bvFTD, while hedonic and emotional motivational changes (anhedonia, emotional apathy) do not seem to play as central a role. As such, our findings suggest that a reduction in spontaneous cognitive activities and accompanying behavioral inertia may be critical harbingers of poor functional outcomes in this syndrome [see also 35].

In contrast, SD patients were characterized largely by motivational impairments related to hedonic processing, with significantly higher levels of anhedonia relative to executive and initiation apathy. Importantly, anhedonia, executive apathy, and initiation apathy emerged as significantly associated with poor functional outcomes in SD. Our findings align with previous research linking apathy to functional decline in SD patients [9] but offer increased precision by pinpointing executive and initiation dimensions of apathy as crucial in this context. Moreover, our findings resonate with recent work highlighting changes in hedonic tone as a prominent yet overlooked feature of the SD motivational phenotype [17–19], and one which has been largely neglected in terms of prognostic outcomes. Recent theoretical frameworks have suggested that loss of hedonic tone in SD might lead to a truncating of interest onto a restricted range of activities that are pursued compulsively, to the neglect of more adaptive behaviors [36]. Our findings of a predominantly anhedonic relative to apathetic profile in SD fit well with this proposal. Rather than displaying a global apathetic profile, carers report SD patients as initiating and executing some aspects of goal-directed behavior, often manifesting in the form of rituals or stereotypical behaviors [36]. Uncovering the nature of these behaviors is an important future direction for this work, ensuring the patient can be supported to remain independent, while also mitigating carer stress and burden. Finally, we note that our current SD sample comprised a mixed group of left- and right-lateralized cases, and it will be important to explore effects of laterality on motivational profiles in SD subtypes, given previous reports of greater anhedonia severity in right SD [17].

Finally, the AD motivational profile was characterized by greater executive and initiation apathy in comparison to anhedonia. This finding resonates with previous reports of less severe emotional apathy [11] and lower levels of anhedonia [16] in AD relative to bvFTD. Importantly, correlation analyses suggested that executive and emotional apathy made the strongest contributions to functional impairment in AD. That executive apathy should be associated with functional decline in AD is not surprising, and potentially reflects widespread difficulties in the cognitive initiating, planning, and implementing of goal-directed actions essential for an array of complex activities of daily living [discussed by 37]. Our finding that emotional apathy is associated with functional decline was somewhat unexpected. It may be that with disease progression, cognitive impairments in AD result in increasing social withdrawal. However, further empirical research will be required to substantiate this proposal. Our findings mesh well with longitudinal studies suggesting that apathy might serve as a behavioral marker of a more aggressive disease course and poorer prognosis in AD [10, 38]. Given that these studies assessed apathy in a unidimensional manner, it will be imperative to chart how the multidimensional nature of apathy changes over the AD disease course and how fluctuations across apathy components relate to different disease trajectories. This is particularly relevant in the context of younger-onset presentations of AD, for whom clinical symptoms may differ from the canonical later-onset presentation [40].

Several methodological considerations warrant discussion. While our emphasis was on multidimensional aspects of motivation, the SHAPS questionnaire provides a somewhat coarse snapshot of anhedonia. Future studies distinguishing between putative dimensions of anhedonia (e.g., anticipatory versus consummatory) will provide important insights in this regard. We acknowledge the need to move beyond traditional carer report questionnaires and to develop objective assays of motivated behaviors as expressed in daily life. This is particularly pertinent given that in the current study we relied on carer report for ratings of patient anhedonia and apathy, while depression symptoms were self-rated by the patient. Notably, early features of anhedonia may be subtle and therefore less amenable to carer report (e.g., loss of interest, decreased pleasure). In addition, there are currently no validated methods for measuring anhedonia in dementia, which highlights the need for multidimensional validated measures in this population. Finally, given all our patient groups were in the “moderate” stage of functional impairment, it will be crucial to systematically track how multidimensional motivational changes vary according to disease staging and dynamically evolve over the disease course. Longitudinal studies with larger sample sizes will enable us to determine whether the phenotypic motivational profiles reported here stabilize or even resolve in each dementia syndrome, with a view to identifying critical periods of transition during which targeted interventions might have optimal efficacy.

## Conclusions

In conclusion, this study provides a fine-grained characterization of distinct motivational phenotypes in dementia and their respective impact on functional outcomes. Motivational disturbances in dementia are gaining increasing attention as predictors of early entry to residential care and patient mortality and are reported as some of the most difficult symptoms to manage by carers [39]. Our findings underscore the importance of screening for apathy and anhedonia in younger-onset dementia populations and delineating the specific stages at which such symptoms emerge. This detailed characterization of motivational phenotypes can inform patient stratification for targeted interventions to potentially improve functional outcomes and reduce carer stress.

### Supplementary Information

Below is the link to the electronic supplementary material.

Supplementary file1 (DOCX 1552 KB)

## Data Availability

The ethical requirement to ensure patient confidentiality precludes public archiving of our data. Researchers who would like to access the raw data should contact the corresponding author who will liaise with the ethics committee that approved the study, and accordingly, as much data that are required to reproduce the results will be released to the individual researcher. No part of the study procedures or analyses were preregistered prior to the research being undertaken.

## Footnotes

1 A large proportion of these data were previously reported in Shaw et al. (2021), with 93% overlap between study samples. Importantly, the original Shaw et al. 2021 study adopted a transdiagnostic approach and did not explore predictors of functional outcomes within specific dementia subtypes.
