# Supplementary material for: Profiles of motivational impairment and their relationship to functional decline in frontotemporal dementia
Source: J Neurol. 2024 May 17;271(8):4963–71. doi: 10.1007/s00415-024-12430-0 (PMC11319612; doi:10.1007/s00415-024-12430-0)
Supplement: Supplementary file 1 — Supplementary file1 (DOCX 1552 KB) [file 415_2024_12430_MOESM1_ESM.docx]

**Profiles of motivational impairment and their relationship to functional decline in frontotemporal dementia**

Siobhán R. Shaw^1,2^, Kristina Horne^1,2^, Olivier Piguet^1,2^, Rebekah M. Ahmed^1,4^, Alexis E. Whitton^5^, Muireann Irish^1,2^*

1. The University of Sydney, Brain and Mind Centre, Sydney, New South Wales, Australia
2. The University of Sydney, School of Psychology, Sydney, New South Wales, Australia
3. Memory and Cognition Clinic, Department of Clinical Neurosciences, Royal Prince Alfred Hospital, Sydney, Australia
4. The University of Sydney, School of Medical Sciences, Sydney, New South Wales, Australia
5. Black Dog Institute, University of New South Wales, Sydney, New South Wales, Australia

*Correspondence: Professor Muireann Irish

Brain and Mind Centre, The University of Sydney,

94 Mallett Street, Camperdown,

Australia, NSW 2050

muireann.irish@sydney.edu.au; Tel.: +61-2-9114-4165

**Files included:**

1. **Supplementary Table 1.** Tests of normality for main study variables of interest
2. **Supplementary Table 2.** Percentage frequency of carer relationship across patient groups.
3. **Supplementary Figure 1**. Pre-morbid ratings of anhedonia severity in dementia.
4. **Supplementary Figure 2.** Pre-morbid ratings of apathy severity in dementia
5. **Supplementary Figure 3.** Motivational profiles in dementia syndromes.
6. **Supplementary Figure 4.** Scatterplots showing associations between anhedonia severity and functional impairment in each dementia group.
7. **Supplementary Figure 5.** Scatterplots showing associations between executive apathy severity and functional impairment in each dementia group.
8. **Supplementary Figure 6.** Scatterplots showing associations between emotional apathy severity and functional impairment in each dementia group.
9. **Supplementary Figure 7.** Scatterplots showing associations between initiation apathy severity and functional impairment in each dementia group.

**Supplementary Table 1**. Tests of normality for main study variables of interest

|  | **bvFTD**  **(n=58)** | **SD**  **(n=30)** | **AD**  **(n=42)** |
| --- | --- | --- | --- |
| Anhedonia | .06 | .57 | .10 |
| Executive apathy | .09 | .19 | .08 |
| Emotional apathy | .26 | .28 | .65 |
| Initiation apathy | .07 | .34 | .07 |
| FRS | .08 | .50 | .39 |

*Note.* Shapiro-Wilks tests of normality were conducted to determine whether data for the main study variables of interest were normally distributed. Anhedonia severity was measured using the Snaith-Hamilton Pleasure Scale (SHAPS) whilst apathy was measured using the Dimensional Apathy Scale (DApS). AD = Alzheimer’s disease; bvFTD = behavioural variant of frontotemporal dementia; FRS = Frontotemporal Dementia Functional Rating Scale; SD = semantic dementia.

**Supplementary Table 2**. Percentage frequency of carer relationship across patient groups.

|  |  | **bvFTD**  **(n=58)** | **SD**  **(n=30)** | **AD**  **(n=42)** |
| --- | --- | --- | --- | --- |
| Carer | Spouse (%) | 80.9 | 75.0 | 83.7 |
|  | Child (%) | 8.8 | 15.6 | 4.7 |
|  | Sibling (%) | 1.5 | 6.3 | 2.3 |
|  | Support worker (%) | - | - | 4.7 |
|  | Other (%) | 8.8 | 3.1 | 4.7 |

*Note.* The ‘Other’ category is a mixed category comprising brother-in-law, sister-in-law, mother, and friend. AD = Alzheimer’s disease; bvFTD = behavioural variant of frontotemporal dementia; SD = semantic dementia.

**Supplementary Figure 1.** Pre-morbid ratings of anhedonia severity in dementia.

**Fig 1.** Premorbid ratings of anhedonia in dementia syndromes as rated by carers on the Snaith-Hamilton Pleasure Scale (SHAPS). Violin plots depict the distribution of data with the width of the shaded area representing the proportion of data located there. Bolded horizontal line depicts the median, while dotted lines depict quartiles. AD = Alzheimer’s disease (n=43); bvFTD = behavioural variant of frontotemporal dementia (n=68); SD = semantic dementia (n=32)

Importantly, a univariate ANCOVA controlling for sex, disease duration, and overall level of cognitive dysfunction on the ACE-III revealed no significant group differences in terms of carer-rated premorbid levels of anhedonia [*F*(2,137)=.34, *p*=.71, $\eta_{p}^{2}$=.005].

**Supplementary Figure 2.** Pre-morbid ratings of apathy severity in dementia

**Fig 2.** Premorbid carer-rated apathy on the Dimensional Apathy Scale (DApS). Violin plots depict the distribution of raw data with the width of the shaded area representing the proportion of data located there. Bolded horizontal line depicts the median. AD = Alzheimer’s disease (n=32); bvFTD = behavioural variant of frontotemporal dementia (n=60); SD = semantic dementia (n=27).

Importantly, the dementia groups did not differ in terms of carer-reported levels of premorbid executive [*F*(2,120)=.88, *p*=.45, $\eta_{p}^{2}$=.022], emotional [*F*(2,120)=.57, *p*=.63, $\eta_{p}^{2}$=.014] or initiation [*F*(2,120)=.11, *p*=.96, $\eta_{p}^{2}$=.003] apathy as determined by separate ANCOVAs controlling for sex, disease duration, and overall level of cognitive dysfunction on the ACE-III.

**Supplementary Figure 3.** Motivational profiles in dementia syndromes.

**Fig 3.** Severity of motivational disturbances in each dementia group. Anhedonia scores were taken from the Snaith Hamilton Pleasure Scale (SHAPS), while apathy subscale scores were derived from the Dimensional Apathy Scale (DApS). All scores are the residual scores (calculated based on patient population before disease onset and current SHAPS and DApS scores). AD = Alzheimer’s disease; bvFTD = behavioural variant of frontotemporal dementia; SD = semantic dementia. Bolded horizontal line depicts the median. Asterisks denote results that emerged as significant in the analyses controlling for sex, disease duration, and overall level of cognitive dysfunction on the ACE-III. **p*<.05, ***p*<.005, ****p*<.001

**Supplementary Figure 4.** Scatterplots showing associations between anhedonia severity and functional impairment in each dementia group.

**Fig 4.** Scatterplots displaying associations between anhedonia severity on the Snaith Hamilton Pleasure Scale (SHAPS) and functional impairment on the Frontotemporal Dementia Functional Rating Scale (FRS) in each patient group. Lower scores denote greater levels of functional impairment on the FRS. All scores are the residual scores (calculated based on patient population before disease onset and current SHAPS). AD = Alzheimer’s disease; bvFTD = behavioural variant of frontotemporal dementia; SD = semantic dementia.

**Supplementary Figure 5.** Scatterplots showing associations between executive apathy severity and functional impairment in each dementia group.

**Fig 5.** Scatterplots showing associations between executive apathy severity and functional impairment on the Frontotemporal Dementia Functional Rating Scale (FRS) in all patient groups. Lower scores denote greater levels of functional impairment on the FRS. Executive apathy subscale scores were derived from the Dimensional Apathy Scale (DApS). All scores are the residual scores (calculated based on patient population before disease onset and current DApS scores). AD = Alzheimer’s disease; bvFTD = behavioural variant of frontotemporal dementia; SD = semantic dementia.

**Supplementary Figure 6.** Scatterplots showing associations between emotional apathy severity and functional impairment in each dementia group.

**Fig 6.** Associations between emotional apathy severity and functional impairment on the Frontotemporal Dementia Functional Rating Scale (FRS) in all patient groups. Lower scores denote greater levels of functional impairment on the FRS. Emotional apathy subscale scores were derived from the Dimensional Apathy Scale (DApS). All scores are the residual scores (calculated based on patient population before disease onset and current DApS scores). AD = Alzheimer’s disease; bvFTD = behavioural variant of frontotemporal dementia; SD = semantic dementia.

**Supplementary Figure 7.** Scatterplots showing associations between initiation apathy severity and functional impairment in each dementia group.

**Fig 7.** Associations between initiation apathy severity and functional impairment on the Frontotemporal Dementia Functional Rating Scale (FRS) in all patient groups. Lower scores denote greater levels of functional impairment on the FRS. Initiation apathy subscale scores were derived from the Dimensional Apathy Scale (DApS). All scores are the residual scores (calculated based on patient population before disease onset and current DApS scores). AD = Alzheimer’s disease; bvFTD = behavioural variant of frontotemporal dementia; SD = semantic dementia.
